# Advances in Understanding Activation and Function of the NLRC4 Inflammasome

**DOI:** 10.3390/ijms22031048

**Published:** 2021-01-21

**Authors:** Balamurugan Sundaram, Thirumala-Devi Kanneganti

**Affiliations:** Department of Immunology, St. Jude Children’s Research Hospital, Memphis, TN 38105, USA; balamurugan.sundaram@stjude.org

**Keywords:** NOD-like receptors, NLR, NLRC4, NAIP, IRF8, inflammasome, cell death, pyroptosis, apoptosis, necroptosis, PANoptosis, PANoptosome, Gram-negative bacteria, *Salmonella*

## Abstract

Innate immune receptors initiate a host immune response, or inflammatory response, upon detecting pathogen-associated molecular patterns (PAMPs) and damage-associated molecular patterns (DAMPs). Among the innate immune receptors, nucleotide-binding oligomerization domain (NOD)-like receptors (NLRs) play a pivotal role in detecting cytosolic PAMPs and DAMPs. Some NLRs can form a multiprotein cytosolic complex known as the inflammasome. Inflammasome activation triggers caspase-1–mediated cleavage of the pore-forming protein gasdermin D (GSDMD), which drives a form of inflammatory cell death called pyroptosis. Parallelly, activated caspase-1 cleaves immature cytokines pro–IL-1β and pro–IL-18 into their active forms, which can be released via GSDMD membrane pores. The NLR family apoptosis inhibitory proteins (NAIP)-NLR family caspase-associated recruitment domain-containing protein 4 (NLRC4) inflammasome is important for mounting an immune response against Gram-negative bacteria. NLRC4 is activated through NAIPs sensing type 3 secretion system (T3SS) proteins from Gram-negative bacteria, such as *Salmonella* Typhimurium. Mutations in NAIPs and NLRC4 are linked to autoinflammatory disorders in humans. In this review, we highlight the role of the NAIP/NLRC4 inflammasome in host defense, autoinflammatory diseases, cancer, and cell death. We also discuss evidence pointing to a role of NLRC4 in PANoptosis, which was recently identified as a unique inflammatory programmed cell death pathway with important physiological relevance in a range of diseases. Improved understanding of the NLRC4 inflammasome and its potential roles in PANoptosis paves the way for identifying new therapeutic strategies to target disease.

## 1. Introduction

Innate immunity is the front-line defense mechanism to protect the host from various pathogenic and sterile insults. The innate immune system is triggered by pathogen-associated molecular patterns (PAMPs), which are components of infectious agents, and damage-associated molecular patterns (DAMPs), which are released during cellular or tissue damage. PAMPs and DAMPs are detected by germline-encoded host sensors called pattern recognition receptors (PRRs) [1,2], and this detection and subsequent response are critical for host survival. Based on their localization, PRRs are classified as membrane-bound PRRs and cytoplasmic PRRs. Membrane-bound PRRs include Toll-like receptors (TLRs) and C-type lectin receptors (CLRs), whereas cytoplasmic PRRs include nucleotide-binding oligomerization domain (NOD)-like receptors (NLRs), absent in melanoma 2 (AIM2)-like receptors (ALRs), and RIG-I-like receptors (RLRs). NLRs recognize a diverse array of ligands which can be from self or non-self, including pathogens. After sensing the ligands, some of the NLR family members assemble an inflammasome, which is a cytosolic multiprotein complex. Inflammasome assembly drives the activation of caspase-1, which processes IL-1β and IL-18 to produce their active forms and cleaves gasdermin D (GSDMD) to trigger a form of inflammatory cell death known as pyroptosis [3,4,5]. Due to their importance in inflammation and host response to pathogens, mutations in NLRs are associated with human autoimmune and autoinflammatory disorders. Although NLR family pyrin domain (PYD)-containing 3 (NLRP3) is the best-characterized inflammasome sensor, several other sensors, including NLRP1, NLR apoptosis inhibitory protein (NAIP)-NLR family caspase recruitment domain (CARD)-containing protein 4 (NLRC4), NLRP6, NLRP9, absent in melanoma 2 (AIM2), and Pyrin, can also form inflammasomes and participate in regulating the host immune and inflammatory response [6,7,8].

**Figure 1:**
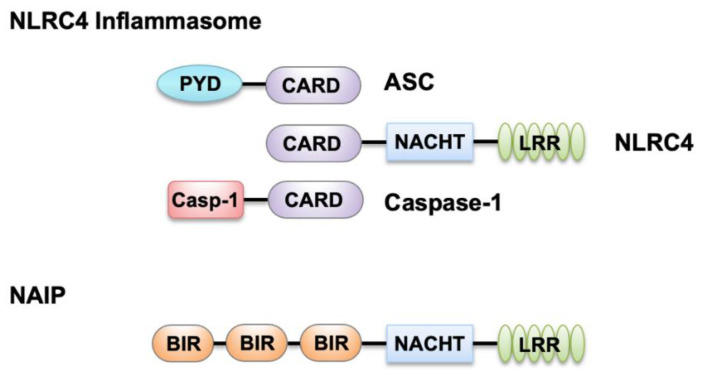
Domains of nucleotide-binding oligomerization domain (NOD)-like receptors (NLR) family caspase recruitment domain-containing protein 4 (NLRC4) inflammasome components and NLR family apoptosis inhibitory proteins (NAIPs). NLRC4 contains an N-terminal caspase recruitment domain (CARD), a central nucleotide-binding domain (NACHT), and a C-terminal leucine-rich repeat (LRR) domain. Both apoptosis-associated speck-like protein containing a CARD (ASC) and caspase-1 contain a CARD in addition to a pyrin (PYD) and caspase-1 (Casp-1) domain, respectively. Like NLRC4, NAIPs also contain a central NACHT and a C-terminal LRR domain, along with N-terminal baculovirus IAP-repeat (BIR) domains.

NLRC4 plays a critical role in detecting Gram-negative bacteria in the cytoplasm, and it was initially discovered and called IPAF (ICE protease-activating factor) for its ability to activate caspase-1 [9]. NLRC4 features a three-domain structure: an amino-terminal CARD, a central nucleotide-binding domain (NACHT), and a carboxy-terminal leucine-rich repeat domain (LRR) (Figure 1).

**Figure 2:**
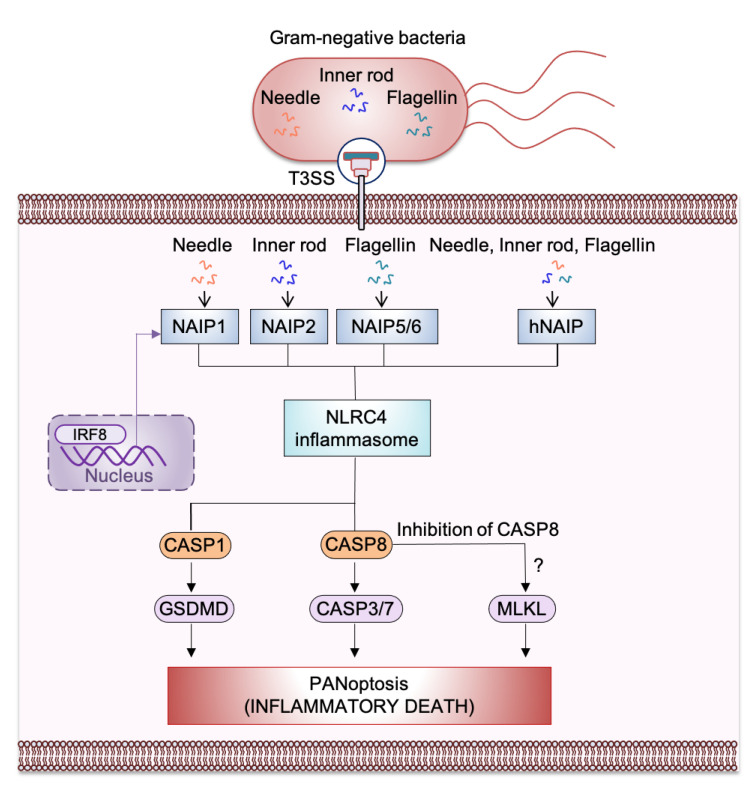
Mechanism of nucleotide-binding oligomerization domain (NOD)-like receptor (NLR) family apoptosis inhibitory protein (NAIP)-NLR family caspase recruitment domain-containing protein 4 (NLRC4) inflammasome-mediated PANoptosis, inflammatory cell death. Ligands from Gram-negative bacteria such as needle and inner rod protein of the Type 3 secretion system (T3SS) can be detected by murine NAIP1 and NAIP2, respectively, whereas flagellin can be detected by murine NAIP5/NAIP6. The single human NAIP (hNAIP) detects all the T3SS proteins such as needle, inner rod protein, and flagellin. After detecting T3SS proteins, NAIP associates with NLRC4 to induce activation of the NAIP/NLRC4 inflammasome. This activation results in inflammasome assembly and the cleavage and activation of caspase-1 (CASP1). Activated CASP1 triggers gasdermin D (GSDMD)-mediated pyroptosis. NLRC4 also activates caspase-8 (CASP8), which goes on to activate caspase-3/-7 (CASP3/7) to trigger apoptosis. Activation of the necroptotic pathway has also been observed during *S.* Typhimurium infection; this may occur through inhibition of CASP8. NAIPs are transcriptionally induced by interferon regulatory factor 8 (IRF8).

NLRC4 can associate with pro–caspase-1 directly through CARD-CARD interactions, which triggers the processing and activation of caspase-1 [10]. Additionally, the adaptor molecule ASC (apoptosis-associated speck-like protein containing a CARD), encoded by the gene *Pycard*, can also facilitate this interaction. Activated NLRC4 can associate with ASC and colocalizes with the ASC-containing speck during *Salmonella* Typhimurium infection [11,12]. ASC contains a PYD and a CARD (Figure 1); its CARD is critical for optimal caspase-1 recruitment into the speck to allow its activation and proteolytic cleavage of pro–IL-1β and pro–IL-18 [11,12,13]. Further, activated caspase-1 drives proteolytic cleavage of the pore-forming protein GSDMD, allowing the N-terminus of GSDMD to oligomerize in the host cell membrane which results in pore formation that causes pyroptosis and the release of cytokines and alarmins (Figure 2). NAIPs act as upstream sensors for NLRC4 inflammasome assembly. They contain an N-terminal baculovirus IAP-repeat (BIR) domain, a central NACHT, and a carboxy-terminal LRR [6] (Figure 1). NAIPs are crucial for detecting bacterial ligands in the cytoplasm, and their association with NLRC4 triggers NAIP-NLRC4 inflammasome activation [6] (Figure 2).

In this review, we will focus on the established functions of NLRC4 along with the most recent findings centered on the role of the NLRC4 inflammasome during infectious and autoinflammatory diseases. We will cover NAIP proteins and their role in ligand detection and NLRC4 inflammasome activation. Finally, we will discuss the role of NLRC4 in cell death and highlight emerging evidence pointing to its potential importance in PANoptosis, which would be interesting to investigate in future studies on inflammasome and cell death biology to identify new strategies to promote host defense while preventing excess inflammation.

## 2. Nucleotide-Binding Oligomerization Domain (NOD)-Like Receptor (NLR) Family Caspase-Associated Recruitment Domain-Containing Protein 4 (NLRC4)—An Innate Cytosolic Sensor

In 2004, researchers first provided genetic proof that *Nlrc4*-deficient bone marrow-derived macrophages (BMDMs) fail to activate caspase-1 and pyroptosis after exposure to *S.* Typhimurium [14]. Later studies identified that *S.* Typhimurium flagellin and flagellin from the Gram-negative bacteria *Legionella pneumonia* can activate NLRC4, which subsequently triggers activation of caspase-1 [15,16,17,18]. Several pathogens are now known to possess flagellin-like virulence factors which can participate in the activation of the NLRC4 inflammasome; examples include *S.* Typhimurium (PrgJ), *Burkholderia pseudomallei* (BsaK), *Escherichia coli* (EprJ and Escl), *Shigella flexneri* (Mxil), and *Pseudomonas aeruginosa* (Pscl) [19]. Additionally, the NLRC4 inflammasome can be activated in certain cases without flagellin during bacteria exposure [16]. Overall, these findings demonstrate that NLRC4 is an important innate immune regulator that detects bacterial virulence factors.

NAIPs act as upstream immune sensor proteins for NLRC4 inflammasome activation. The murine genome encodes a total of seven NAIPs whereas the human genome encodes only one (hNAIP) [20]. In mice, NAIP5 and NAIP6 can activate NLRC4 specifically in response to bacterial flagellin [21]. NAIP5 directly interacts with flagellin and promotes the physical NAIP5-NLRC4 association which triggers NLRC4 inflammasome activation [21,22]. The related NAIP2 functions similarly to NAIP5 and serves as a specific inflammasome receptor for type III secretion system (T3SS) rod proteins such as *S.* Typhimurium PrgJ and *B. pseudomallei* BsaK [22]. hNAIP is functionally similar to murine NAIP1 in that they both sense the T3SS needle protein [23,24]. Furthermore, hNAIP can trigger NLRC4 inflammasome activation in response to both T3SS components and flagellin [25,26]. CprI, a *Chromobacterium violaceum* T3SS protein, is specifically recognized by hNAIP to trigger NLRC4 inflammasome activation [22].

The expression of NAIPs and NLRC4 are regulated by the interferon regulatory factor 8 (IRF8) transcription factor during bacterial infection [27] (Figure 2). IRF8 has been shown to be specifically required for activation of the NLRC4 inflammasome in BMDMs during *S.* Typhimurium, *Burkholderia thailandensis*, or *P. aeruginosa* infection. In vivo, *Irf8*-deficient mice are highly susceptible to bacterial infection compared with wildtype animals, confirming the critical role of IRF8 in NAIP/NLRC4 inflammasome activation [27]. Altogether, NAIPs act as an upstream sensor molecule that helps to recognize cytosolic PAMPs for NLRC4 inflammasome activation.

## 3. Role of NLRC4 in Host Defense

The innate immune function of the NLRC4 inflammasome has been extensively studied during bacterial infections, especially the foodborne bacterium *S.* Typhimurium. Studies have documented that *Nlrc4*-deficient animals are more susceptible to *S.* Typhimurium infection and have increased bacterial loads in the cecum, liver, and spleen compared with control animals [11,28,29,30,31], suggesting that NLRC4 is indispensable to protect the host from *S.* Typhimurium infection. However, similar studies have demonstrated that *Nlrc4* knockout mice have no difference in bacterial load compared to wildtype mice after *S.* Typhimurium infection [13,31,32]. Functional redundancies between NLRC4 and NLRP3 could be the reason for these conflicting results. Indeed, later studies have shown that *Nlrc4* and *Nlrp3* double knockout mice are highly susceptible to *S.* Typhimurium and have increased bacterial loads in the spleen, liver, and mesenteric lymph nodes compared with control animals [13]. Other potential factors that could be contributing to the conflicting results observed in studies of NLRC4 during *S.* Typhimurium infection include differences in the bacterial strain used for infection, the route of bacterial administration, the genetic background of the mice used, and the gut microbiota of mice housed in different animal facilities. The intricate relationship between the gut microbiota and inflammasome activation has made it clear that using littermate controls is essential for obtaining reliable results from in vivo experiments [33,34]. Littermates are preferable to antibiotic depletion of the native microbiome, as treating mice with antibiotics can cause gut injury which was found to be associated with sepsis-like disease and the systemic spread of a multidrug-resistant *E. coli* pathobiont which can activate the NAIP5-NLRC4 inflammasome [35], further confounding results. Despite these conflicting findings, it is clear that NLRC4 is important in sensing bacterial ligands during *S.* Typhimurium infection [11,28,29,30,31].

Similar to *S.* Typhimurium, another enteric bacterial pathogen *Citrobacter rodentium* causes increased pathology and hyper-susceptibility in mice deficient in *Nlrp3*, *Nlrc4*, and *Casp1*, suggesting a critical role for NLRC4 in host defense against *C. rodentium* [36]. In addition to the role of NLRC4 in immune cells from the hematopoietic compartment during this infection, NLRC4 activation in the non-hematopoietic cellular compartment also occurs, especially in gut epithelial cells. Transplanting wildtype bone marrow cells to *Nlrc4*^−/−^ mice using a bone marrow chimera technique does not control the *C. rodentium* pathogen load, suggesting NLRC4 is important in the non-hematopoietic compartment [37]. Follow-up studies confirmed the essential functions of NLRC4 in the non-hematopoietic compartment by using tissue-specific gene knockout mice. Similar results have been observed with other pathogens. For example, mouse gut epithelial-specific deletion of *Naip1–6* leads to an increased pathogen load after *Salmonella* infection [29]. Later studies showed that the expulsion of infected enterocytes relies on NLRC4 by using gut epithelium-specific *Nlrc4* knockout mice [30].

Apart from its importance in the host response to these enteric pathogens, NLRC4 also plays a crucial role in eliminating non-enteric bacteria. The NAIP-NLRC4 inflammasome can be activated by the flagellated pneumonia-causing bacteria *Legionella pneumophila*. *Nlrc4*-deficient mice fail to clear *L. pneumophila* upon nasal infection, while wildtype mice can clear the pathogen [38,39,40,41]. Similar to the importance of NLRC4 during infection with *L. pneumophila, Nlrc4*-deficient mice infected with other *Legionella* species, including *L. micdadei, L. bozemanii, L. gratiana, and L. rubrilucens,* are more susceptible and have increased bacterial burden compared with wildtype infected mice [39,40,41,42]. Later studies have also demonstrated the importance of NLRC4 during *P. aeruginosa* infection. *Nlrc4*-deficient macrophages are markedly resistant to *P. aeruginosa*-induced cell death and have reduced secretion of IL-1β. *P. aeruginosa* isolates express the effector molecule exoenzyme U (ExoU), which is capable of inhibiting caspase-1–driven proinflammatory cytokine production [43]. Upon exposure to *P. aeruginosa*, *Nlrc4*-deficient mice have an increased bacterial burden in the bronchoalveolar lavage, but not in lung tissues when compared to control animals [44].

In contrast to these protective roles of NLRC4 during several Gram-negative bacterial infections, NLRC4 can also contribute to pathogenesis. *Helicobacter pylori* can cause chronic infection and lead to gastric ulcers and gastric adenocarcinomas. NLRC4 is crucial for IL-18 production from both human and murine gastric epithelial cells upon exposure to *H. pylori*. *Nlrc4*-deficient mice have reduced inflammation and control the bacterial burden more successfully than wildtype infected mice do [45], highlighting the pathogenic role NLRC4 can play during infection.

Overall, these studies show that the NAIP-NLRC4 inflammasome has dual roles during bacterial infection. NLRC4 protects the host against certain pathogens, such as *Salmonella*, *Citrobacter* or *Legionella*, but its activation may also be pathogenic by triggering strong inflammation during some infections, such as during *Helicobacter* infection.

## 4. Role of NLRC4 in Autoinflammatory Diseases

While NLRC4 activation is critical for activating the immune response and driving inflammation during bacterial infection, its overactivation can cause aberrant cell death and cytokine release. Therefore, mutations that cause hyperactivation of NLRC4 are potentially deleterious and can cause autoinflammatory disease. A de novo missense mutation (c.1009A > T, encoding p.Thr337Ser) affecting *NLRC4* causes early-onset recurrent fever flares and macrophage activation syndrome (MAS). This mutation can trigger constitutive activation of caspase-1 along with increased production of IL-1β and IL-18 in patient-derived and mutant *NLRC4*-transduced macrophages [46]. Another de novo gain-of-function mutation in *NLRC4* causes a p.Val341Ala substitution in the helical domain 1 (HD1), which causes constitutive inflammasome activation in syndromes like neonatal-onset enterocolitis, periodic fever, and fatal or near-fatal episodes of autoinflammation [47]. Additionally, familial cold autoinflammatory syndrome (FCAS) in one Japanese family was found to be due to a missense mutation in *NLRC4* causing enhanced activation of caspase-1 along with increased production of IL-1β. Expression of this mutant *Nlrc4* in mice causes severe dermatitis, arthritis, and splenomegaly along with augmented infiltration of neutrophils and cold-induced exanthema [48]. A case study identified a novel mutation in the LRR domain of *NLRC4* (c.G1965C, p.W655C) which contributes to autoinflammatory disease. The p.W655C *NLRC4* mutation causes an increase in ASC speck formation and caspase-1–mediated IL-1β and IL-18 production, eventually causing increased cell death [49]. Another report identified a mutation in the LRR domain of *NLRC4* that is associated with recurrent fever, skin erythema, and inflammatory arthritis symptoms [50].

Due to the pathogenic role of cytokine signaling in diseases associated with *NLRC4* mutations, clinical blockade of these pathways has been pursued as a therapeutic strategy. Anakinra is an IL-1 receptor antagonist (IL-1Ra), and it is United States Food and Drug Administration (FDA) approved to treat certain inflammatory diseases [51]. Studies have shown that the combination of anakinra and rapamycin treatment could benefit patients with *NLRC4* mutations [52]. Rapamycin has been used as an adjuvant to potentiate the action of anakinra. When given to an anakinra-treated patient, rapamycin resulted in reduced secretion of IL-18 along with a reduction in C-reactive protein and ferritin [52]. Recombinant IL-18 binding protein treatment also improves outcomes in patients with *NLRC4* mutations who have MAS symptoms [53]. Future studies will continue to improve our ability to target molecules in the NLRC4 inflammasome activation cascade to provide new treatment strategies for patients.

## 5. Role of NLRC4 in Cancer

Aberrant activation of the inflammasome plays a major role in different stages of tumor progression including immunosuppression, proliferation, angiogenesis, and metastasis. Conversely, inflammasome activation can trigger tumor suppression by maintaining intestinal barrier integrity, suggesting that the inflammasome has dual roles in cancer development [54]. In 2010, studies showed the importance of NLR inflammasomes in colitis-associated cancer (CAC) by using animals deficient in inflammasome components, including *Pycard*, *Casp1*, *Nlrp3*, and *Nlrc4* [55,56,57]. Mice lacking *Pycard* and *Casp1* are highly susceptible to disease, morbidity, and polyp formation which are correlated with decreased levels of IL-1β and IL-18 at the tumor site [55]. *Nlrp3*-deficient mice also have CAC pathology; however, the disease outcome is less severe in *Nlrp3^−/−^* mice compared with *Asc^−/−^* or *Casp1^−/−^* animals, suggesting another inflammasome is also involved in reducing disease burden [55]. While this study identified no significant difference in CAC pathology or disease outcome between *Nlrc4^−/−^* and control animals [55], a parallel study found that treatment of *Nlrc4^−/−^* mice with the DNA damage-inducing compound azoxymethane (AOM) and dextran sulfate sodium (DSS) causes enlarged tumor volume, reduced apoptosis and enhanced colonic epithelial cell proliferation [58]. Additionally, *Nlrc4^−/−^* mice are more susceptible to DSS-induced colitis compared with wildtype control mice [59]. Collectively, these findings indicate a crucial role for NLRC4 in protecting the gut.

On a cellular level, cytokines and chemokines are critical for tumor killing, and NLRC4 activation is essential for cytokine and chemokine production in tumor-associated macrophages. NLRC4 is necessary for the generation of IFN-γ–producing CD4^+^ and CD8^+^ T cells in the murine model of B16F10 melanoma [60]. However, another recent study demonstrated that NLRC4 has no role in the progression of melanoma, with no difference observed in the tumor incidence between littermate wildtype and *Nlrc4^−/−^* mice [28]. Alternatively, obesity-associated NLRC4 inflammasome activation along with IL-1 signaling trigger breast cancer progression [61]. The disease progression is driven primarily through vascular endothelial growth factor A (VEGFA) expression and angiogenesis. Furthermore, metformin treatment was found to inhibit obesity-associated tumor progression [61].

Overall, NLRC4 has been found to play contradictory roles in cancer progression; this may be due to lack of littermate controls in in vivo studies, differences in the gut microbiota between different animal facilities, differences in the genetic background of the mouse lines, and differences in the cancer models and experimental techniques. Future studies will be needed to clarify the function of NLRC4 in cancer using littermate controls.

## 6. Role of NLRC4 in Pyroptosis, Apoptosis, Necroptosis, and PANoptosis

Activation of different programmed cell death pathways, including pyroptosis, apoptosis, and necroptosis, is essential for clearing pathogens from the host upon infection. Pyroptosis and necroptosis are lytic, immunologically active forms of cell death, whereas apoptosis has historically been considered an immunologically silent cell death pathway; however more recent evidence suggests it is not always silent [62]. Recent work has identified that several infectious agents and sterile insults can induce inflammatory cell death through PANoptosis [63]. PANoptosis is a unique, physiologically relevant, inflammatory programmed cell death pathway activated by specific triggers and regulated by the PANoptosome, a molecular scaffold for contemporaneous engagement of key molecules from pyroptosis, apoptosis, and necroptosis [63,64,65,66,67,68,69,70,71,72,73,74,75,76,77]. Biochemical characterization has shown interactions between PANoptotic molecules [63]. Immunoprecipitation of NLRP3 confirmed that Z-DNA-binding protein 1 (ZBP1), receptor-interacting serine/threonine-protein kinase 3 (RIPK3) and receptor-interacting serine/threonine-protein kinase 1 (RIPK1) can interact with NLRP3. Similarly, immunoprecipitation of RIPK3 resulted in the co-immunoprecipitation of caspase-8, ASC, RIPK1, NLRP3, and ZBP1, suggesting that there are direct interactions between PANoptotic molecules to form a PANoptosome complex [63]. The synergy and crosstalk between these molecules, which were previously thought to be dedicated to their representative cell death pathways, is critical to modulate several inflammatory and infectious diseases and cancer.

While PANoptosis has most often been described in conjunction with NLRP3 inflammasome activation to date [63,68,69,70,71,72,73,74,75,76,77], multiple lines of evidence suggest that NLRC4 may also be playing a critical role in PANoptosis. Direct crosstalk between NLRC4 and apoptosis has been known for many years. Caspase-mediated cleavage of the DNA damage sensor poly (ADP-ribose) polymerase 1 (PARP1) is a hallmark of apoptosis. Activation of the NLRP3 and NLRC4 inflammasomes induces processing of full-length PARP1 into a fragment of 89 kDa. Macrophages deficient in *Casp1, Nlrp3, Nlrc4,* or *Pycard* cannot cleave PARP1, suggesting that protease-mediated inactivation of PARP1 is a shared feature of apoptotic and pyroptotic cell death pathways [65]; these finding laid the early foundation for the concept of PANoptosis.

Recently, a study showed that *S.* Typhimurium infection induces PANoptosis [63]. A subsequent study confirmed that there is an interconnection between pyroptosis, apoptosis, and necroptosis cell death pathways during *S.* Typhimurium infection [78]. Because *S*. Typhimurium induces PANoptosis and previous studies have clearly shown that the NAIP/NLRC4 inflammasome is activated during *Salmonella* infection, it is highly likely that the NAIP/NLRC4 inflammasome contributes to PANoptosis during bacterial infection. A recent study also identified activation of caspase-1 and GSDMD (pyroptosis), caspase-8, caspase-7, and caspase-3 (apoptosis), and mixed lineage kinase domain-like pseudokinase (MLKL) (necroptosis) during *Salmonella* infection [63]. Combined deletion of the PANoptotic molecules *Casp1*, *Casp11*, *Ripk3*, and *Casp8* protects macrophages from cell death during *S.* Typhimurium infection. However, deleting individual cell death components does not completely protect against cell death, suggesting a crucial role of PANoptosis during *S.* Typhimurium infection [63]. Further experimental evidence is needed to clarify the association of NAIP/NLRC4 with the molecules of the PANoptosome complex and to fully understand the role of NLRC4 in PANoptosis for host defense mechanisms (Figure 2).

## 7. Summary and Future Perspectives

Research on the NAIP/NLRC4 inflammasome has progressed drastically in the last decade, especially with regard to the role of NLRC4 as an innate immune sensor, the importance of NAIPs in sensing to give NLRC4 its specificity, and the role of NLRC4 in autoinflammatory diseases and cancer.

Studies on NLRC4 identified that *S.* Typhimurium-infected BMDMs can trigger multiple cell death pathways [63,78]. Cell death is an important phenomenon to clear pathogens and allow host survival. Several studies have documented crosstalk between cell death pathways involving NLRC4 during infection and inflammatory conditions. In intestinal epithelial cell-specific *Nlrc4*-deficient mice, caspase-1 and GSDMD are not essential for cell death, whereas caspase-1 and caspase-8 are necessary for cell expulsion during *Salmonella* infection [30]. This study supports the findings that the NLRC4 inflammasome can recruit caspase-8, a key PANoptosome component, by interacting with the PYD of ASC and death effector domain of caspase-8 [12,79,80]. Additionally, NLRP1b and NLRC4 trigger caspase-8–mediated apoptosis as an alternative cell death program in *Casp1*-deficient macrophages and intestinal epithelial organoids, providing further evidence for the crosstalk between these pathways. The caspase-8 adaptor FADD is recruited to ASC specks, which serve as cytosolic platforms for caspase-8 activation and NLRP1b/NLRC4-induced apoptosis [81]. Similarly, during *B. pseudomallei* infection, the apoptotic caspase-7 is activated downstream of the NLRC4 inflammasome and caspase-1 and requires caspase-9 processing. Therefore, the initiation of different cell death pathways seems to be an effective strategy to limit intracellular *B. pseudomallei* infection [82]. Furthermore, in an ischemic stroke model, the NLRC4 inflammasome mediates pyroptotic and apoptotic cell death in microglial cells [83]. In addition to pyroptosis and apoptosis, a study demonstrated that necroptosis can also contribute to the host immune response during *S.* Typhimurium infection [84]. Mechanistically, type I interferon-mediated necroptosis occurs in macrophages during *S.* Typhimurium infection, triggering an increased persistence of bacterial load. However, in the absence of the necroptotic molecule RIPK3, there is enhanced control of *S.* Typhimurium in vivo [84].

Overall, accumulating evidence suggests that crosstalk occurs between multiple cell death pathways involving NLRC4 in both infection and inflammatory diseases. Future research is needed to answer crucial questions such as how NLRC4 regulates PANoptosis, what are all the critical components involved in each cell death pathway and is there any specific master regulator which connects multiple cell death pathways during NLRC4 activation. Additionally, given that both protective and pathogenic roles of NLRC4 have been observed to date, it will be important to identify the specific infections and inflammatory conditions in which PANoptosis is beneficial vs detrimental. These answers will allow for the identification of new targets and therapeutic strategies to optimize NLRC4 activation for host defense while preventing autoinflammatory disease.
